# Limited evidence for diagnosing bacterial skin infections in older adults in primary care: systematic review

**DOI:** 10.1186/s12877-019-1061-y

**Published:** 2019-02-18

**Authors:** Oghenekome A. Gbinigie, José M. Ordóñez-Mena, Thomas Fanshawe, Annette Plüddemann, Carl J. Heneghan

**Affiliations:** Nuffield Department of Primary Care Health Sciences, University of Oxford, Radcliffe Primary Care Building, Radcliffe Observatory Quarter, Woodstock Road, Oxford, OX2 6GG UK

**Keywords:** Bacterial infections, Skin infections, Signs and symptoms, Older adults, Diagnosis, primary health care

## Abstract

**Background:**

Older adults with bacterial skin infections may present with atypical symptoms, making diagnosis difficult. There is limited authoritative guidance on how older adults in the community present with bacterial skin infections. To date there have been no systematic reviews assessing the diagnostic value of symptoms and signs in identifying bacterial skin infections in older adults in the community.

**Methods:**

We searched Medline and Medline in process, Embase and Web of Science, from inception to September 2017. We included cohort and cross-sectional studies assessing the diagnostic accuracy of symptoms and signs in predicting bacterial skin infections in adults in primary care aged over 65 years. The QUADAS-2 tool was used to assess study quality.

**Results:**

We identified two observational studies of low-moderate quality, with a total of 7991 participants, providing data to calculate the diagnostic accuracy of 5 unique symptoms in predicting bacterial skin infections. The presence of wounds [LR+: 7.93 (CI 4.81–13.1)], pressure sores [LR+: 4.85 (CI 2.18–10.8)] and skin ulcers [LR+: 6.26 (CI 5.49–7.13)] help to diagnose bacterial skin infections. The presence of urinary incontinence does not help to predict bacterial skin infections (LR + ‘s of 0.99 and 1.04; LR-‘s of 0.96 and 1.04).

**Conclusions:**

Currently, there is insufficient evidence to inform the diagnosis of bacterial skin infections in older adults in the community; clinicians should therefore rely upon their clinical judgement and experience. Evidence from high quality primary care studies in older adults, including studies assessing symptoms traditionally associated with bacterial skin infections (e.g. erythema and warmth), is urgently needed to guide practice.

**Electronic supplementary material:**

The online version of this article (10.1186/s12877-019-1061-y) contains supplementary material, which is available to authorized users.

## Background

Life expectancy is increasing, resulting in a growing population of older people. People aged over 65 years make up approximately one sixth of the population, but account for one in three outpatient attendances [1]. Due to a combination of factors including immunosenescence, that is age-related immune dysfunction and inability to mount an adequate response to pathogenic insults [2] and age-related thinning of the skin [3], the skin of older adults is more susceptible to bacterial infections. Soft tissue and skin infections such as cellulitis are common in older adults [4]. If these conditions are not treated promptly, sepsis may ensue. Each year in the United Kingdom (UK), there are 150,000 cases of sepsis in the population, causing 44,000 deaths [5].

Cellulitis is the fourth leading cause of emergency hospital admissions in the UK for acute ambulatory care sensitive conditions (ACSCs); that is, conditions that could effectively be managed in the community [6]. Emergency hospital admissions for patients over the age of 65 years with ACSCs is on the rise [7]. The reasons for the escalation in hospital admissions of older patients with these conditions are not clear, however, bacterial infections frequently present in an atypical fashion in older adults, which can create a diagnostic conundrum for clinicians [8]. Symptoms and signs that are typically associated with bacterial infections in younger adults, such as fever, might be absent in older people [9]. Poor vascular supply, more common in old age, can reduce the signs and symptoms of erythema, warmth and tenderness that are typically associated with cellulitis [10, 11]. Much of the research that has been performed in this area has been conducted in secondary care rather than in primary care.

There is a paucity of official guidance to aid clinicians in illuminating these non-classical presentations. There are no specific NICE or SIGN guidelines relating to the clinical features of bacterial skin infections in older adults.

In order to effectively diagnose and treat bacterial skin infections in older adults in the primary care setting, a clearer understanding of the clinical features that are predictors of infection in this age group is required. Once established, further appropriate management can be initiated, with the goal of reversal of morbidity, and avoidance of premature mortality. The aim of this systematic review is to determine the clinical features that help to diagnose bacterial skin infections (referred to as ‘skin infections’ from here onwards) in older adults in the community.

## Methods

The methods used are similar to those published in a previous systematic review [8].

### Search strategy

Electronic searches were conducted in Medline and Medline in process, Embase and Web of Science, from inception up to February 2016 and searches were updated in September 2017 (See Additional file 1 for full search strategy). Google Scholar was also searched for relevant internet proceedings, and we hand searches the bibliography of retrieved full texts. Two reviewers (OAG and JMOM) independently determined eligibility of articles with disagreements were resolved through discussion, or the opinion of a third reviewer was sought (TRF).

### Inclusion criteria

We included studies of cohort and cross-sectional design, providing information to assess the diagnostic accuracy of symptoms and/or signs in predicting skin and soft tissue infections (including cellulitis) and providing a reference standard for confirming the diagnosis. We included studies conducted in the outpatient setting in patients over 65 years old, although we also included studies in which a small proportion of participants were aged under 65 years. Included studies had to provide sufficient data to enable construction of two by two tables.

### Exclusion criteria

As described elsewhere [8], we excluded studies conducted in immunosuppressed participants; conducted in developing countries; not published in English and with non-human subjects. Systematic reviews, case reports, case series and conference abstracts were similarly excluded. We excluded studies conducted in Accident and Emergency (A&E) units as the prevalence of serious disease in A&E is likely to be higher than in outpatient settings outside of hospital [12].

### Quality assessment

The quality of included studies was independently assessed by two reviewers [OAG and JMOM] using the Quality Assessment of Diagnostic Accuracy Studies-2 (QUADAS-2) tool [13] with disagreements resolved through discussion, or seeking the opinion of a third assessor (AP).

### Data extraction and analysis

Data was extracted or derived from available information independently by two reviewers (OAG and JMOM) from individual studies into two by two tables. Any discrepancies were resolved through discussion, or the opinion of a third reviewer was sought (TRF). Diagnostic accuracy measures, including positive (+) and negative (−) likelihood ratios (LRs) and pre- and post-test probabilities of disease, were calculated for each symptom or sign in relation to skin infection.

The results have been presented in narrative format and on a dumbbell plot derived in Microsoft Excel (Redmond, WA) [14], showing LR+ and LR- with 95% confidence intervals (CI) and the pre-test and post-test probabilities of skin infection given presence or absence of a particular symptom or sign. Symptoms or signs with a statistically significant LR+ or LR- were deemed potentially helpful rule in or rule out tests, respectively [8].

## Results

**Fig. 1:**
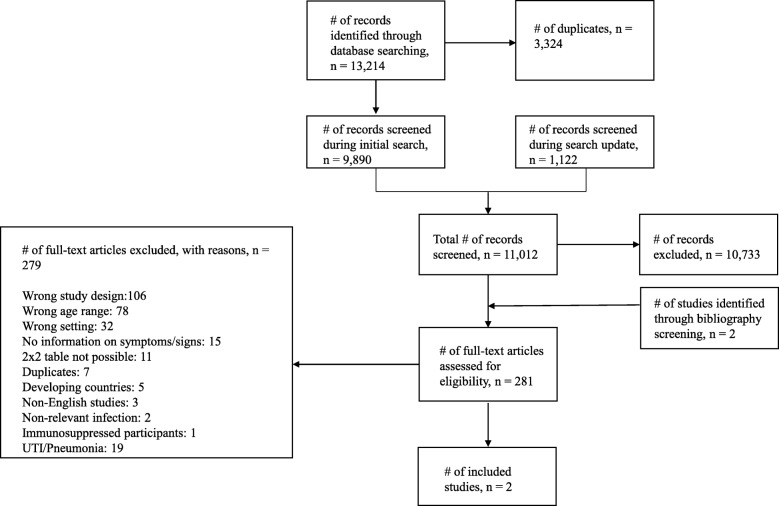
Flow chart showing the process for identification of studies eligible for inclusion

Our initial search in February 2016 identified 9890 non-duplicate results and an additional 1122 non-duplicate results were identified when the search was updated in September 2017. 281 eligible studies were identified through title and abstract and bibliography screening, and after full text screening two studies met the inclusion criteria (Fig. 1). We included in our search strategy all bacterial infections that were of interest (see Additional file 1). Nineteen studies [15–33] focused on urinary tract infection and pneumonia and are therefore not included in this analysis. Table 1 shows details of the two included cross-sectional studies, including 7991 participants [34, 35]. One study [34] was conducted in German nursing homes in 2012 and the other [35] in American nursing homes and long term care facilities in 1991. In both studies, skin infections were not the only infections being investigated. The prevalence of skin infection was very low in both studies at under 2%.

**Table 1:** Characteristics of included studies

Author, year and country	Study type	Study setting	Number of participants	Age (years)	Index tests	Reference Test
Heudorf et al. 2012 Germany [34]	Cross-sectional	Nursing homes	3732	11% under 65 years	Incontinence Disorientation Wounds Pressure sores	Adapted McGeer criteria; physician diagnosis of infection was included as a criterion to avoid under-estimation of the infection rate due to lack of on-site diagnostic testing.
Magaziner et al. 1991 USA [35]	Cross-sectional	Long term care facilities/Nursing homes	4259	> 65	Incontinence Skin ulcer	A combination of symptoms/signs/lab investigations. Not all patients had a bacterial skin culture.

**Fig. 2:**
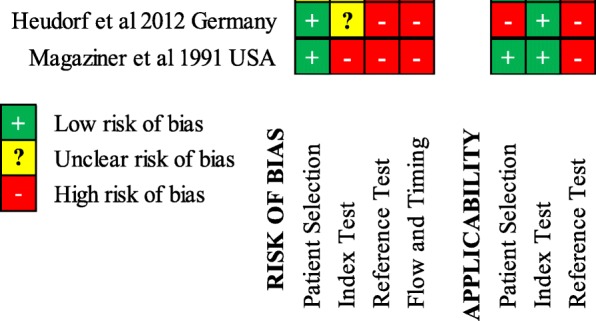
Risk of bias summary. QUADAS-2 Risk of bias and applicability summary showing review authors’ judgements about each domain for included studies

**Fig. 3:**
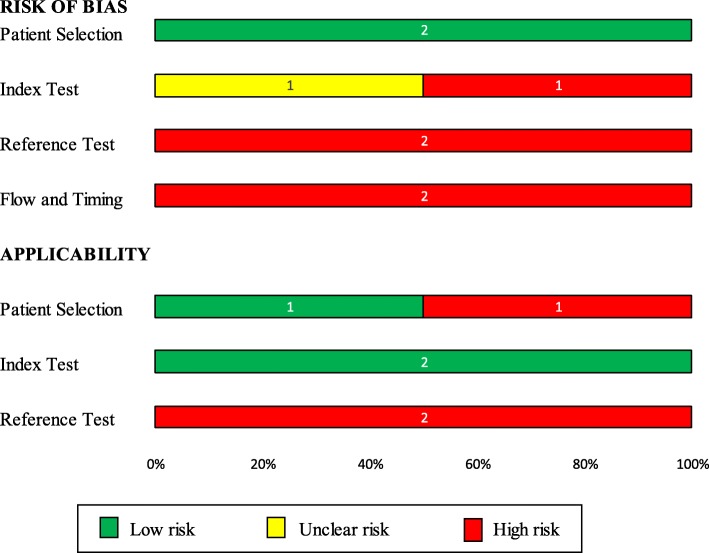
Risk of bias graph**.** QUADAS-2 Risk of bias and applicability graph showing review authors’ judgements about each domain across the included studies

Both studies were of low-moderate quality (Figs. 2 and 3) using the QUADAS-2 tool [13]. Both studies suffered significant delays between assessment of the index test (symptoms/signs) and the reference test, and not all participants in either study had a definitive reference test performed.

**Fig. 4:**
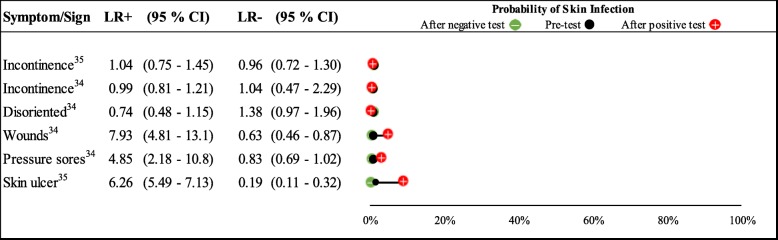
Likelihood ratios and probability plot for symptoms and signs in predicting skin infections. Likelihood ratios and pre- and post-test probabilities for symptoms and signs in predicting skin infections. Positive and negative likelihood ratios with 95% confidence intervals are presented for each symptom. The black dot within the dumbbell plot represents the pre-test probability of infection (i.e. prevalence). The red dot represents the probability of infection after a positive test (i.e. given that the symptom is present), and the green dot represents the probability of infection after a negative test (i.e. given that the symptom is absent)

The two studies report six estimates of five unique symptoms and signs (Fig. 4 and Additional file 2). Of the symptoms/signs reported, the presence of wounds [LR+: 7.93 (95% CI 4.81–13.1)], pressure sores [LR+: 4.85 (95% CI 2.18–10.8)] and skin ulcers [LR+: 6.26 (95% CI 5.49–7.13)], were all predictors of skin infections. The absence of skin ulcers and wounds [LR-: 0.19 (95% CI 0.11–0.32) and 0.63 (95% CI 0.46–0.87), respectively)], but not the absence of pressure sores [LR-: 0.83 (95% CI 0.69–1.02)], helped to rule out a diagnosis of skin infection. Being disoriented did not help to make a diagnosis of a skin infection.

One symptom (incontinence) was assessed by both studies [34, 35]. Neither study found that incontinence was helpful in ruling in, or out, skin infections [LR + ‘s: 0.99 (0.81–1.21) and 1.04 (0.75–1.45); LR-‘s: 1.04 (0.47–2.29) and 0.96 (0.72–1.30)].

Due to the low prevalence of skin infection in the included studies, the post-test probability of infection was not raised higher than 9% (by presence of skin ulcers) for any symptom or sign investigated.

## Discussion

### Main findings

The evidence from studies of predictors of skin infections in older outpatients suggests that the presence of pressure sores, wounds and ulcers are helpful predictors, and the absence of skin ulcers and wounds help to rule-out skin infections. Being disoriented and urinary incontinence do not help to diagnose skin infections. This data comes from a limited number of studies of low to moderate quality.

Wounds, pressure sores and skin ulcers were all found to be predictors of skin infection; this would seem intuitive as each presents a portal of entry for bacteria. The estimates obtained for incontinence in predicting skin infections both showed that incontinence does not help to predict skin infection [34, 35]. We hypothesize that this could be a result of incontinence leading to more attention being paid to skin hygiene. Another theory is that urine has an anti-bacterial effect due to urea content [36]. Further studies of high quality are needed to confirm or refute this.

### Strengths and limitations

To the best of our knowledge, this is the first systematic review assessing the utility of symptoms and signs in diagnosing skin infections in older adults in the outpatient setting. We employed a robust and broad search strategy to identify studies. When necessary, we contacted the authors of studies to clarify details in the papers being screened for eligibility. All data was dual extracted. However, due to the breadth of the review, it is likely that we may not have identified all pertinent studies, especially unpublished studies. To address this, we plan to update the review in light of new evidence.

Due to the small number of included studies that are of low-moderate quality, our results should be interpreted with caution. Furthermore, we were only able to extract data for a limited number of symptoms and signs, and only one symptom was common to both studies. Whilst we determined that some symptoms and signs were statistically significant based on LRs, these same symptoms and signs may not necessarily translate into clinical significance for making a diagnosis. None of the symptoms assessed increased the probability of skin infection beyond 9%, and therefore may be insufficient to rule in the diagnosis with certainty. This reinforces the importance of interpreting the LRs in the context of prior probability (see probability plots).

We also recognise that there is no single gold standard test for diagnosing skin infections; clinicians tend to combine clinical assessment with tests (such as cultures). Incorporation bias is therefore difficult to avoid and can cause an overestimation of the accuracy of a given index test. A symptom or test in isolation is unlikely to be sufficient to rule in or out a diagnosis of skin infection.

One of the studies included a small percentage of the total participants under the age of 65 years [34]. In this study, 11% of participants were under 65 years of age, however, the author informed us that the two by two table data remained essentially unchanged after excluding them. We can therefore be confident that this would not have biased the obtained estimates.

Finally, we excluded non-English studies and may therefore have omitted studies otherwise suitable for inclusion.

### Implications for future research

More high quality diagnostic studies in the outpatient setting are required to allow for meaningful meta-analysis of the data and robust conclusions to be made. It would be helpful if these studies also assessed the ability of symptoms commonly associated with skin infections (e.g. warmth and erythema) as predictors in older adult outpatients. Studies assessing the utility of combinations of different symptoms in making a diagnosis of skin infections would be beneficial. Increasing the evidence base in this area could facilitate creation of evidence-based authoritative guidance and the generation of clinical prediction rules to help minimise the uncertainty of outpatient clinicians when assessing older adults.

### Implications for clinical practice

The limited evidence of low to moderate quality appraising the utility of symptoms and signs in diagnosing skin infections in older adults in primary care suggests that the presence of wounds, pressure sores and skin ulcers are predictors of skin infection, and that the absence of skin ulcers and wounds help to rule-out skin infections. However, the diagnosis of bacterial skin infections based only on the presence of wounds, pressure sores or skin ulcers, even in the presence of a positive culture, would increase the risk of giving unnecessary antimicrobial treatment and inducing antibiotic resistance. Urinary incontinence and being disoriented do not help to diagnose skin infections. Further, high quality studies with large numbers of participants need to be performed in this area to corroborate our findings and guide clinical practice.

## Conclusions

At present, there is insufficient evidence to inform the diagnosis of bacterial skin infections in older adults in primary care. Until further evidence from high quality studies conducted in this area can be evaluated, clinicians must ultimately rely upon their own clinical judgement and experience.

## Additional files

Additional file 1: Search strategy. (DOCX 23 kb)

Additional file 2: **Table S1.** Two-by-two table data for symptoms and signs in relation to skin infections. (PPTX 38 kb)

## Abbreviations

CI: Confidence Interval
LR-: Negative likelihood ratio
LR+: Positive likelihood ratio
QUADAS-2: Quality assessment of Diagnostic Accuracy Studies − 2
