# Supplementary material for: Limited evidence for diagnosing bacterial skin infections in older adults in primary care: systematic review
Source: BMC Geriatr. 2019 Feb 18;19:45. doi: 10.1186/s12877-019-1061-y (PMC6380032; doi:10.1186/s12877-019-1061-y)
Supplement: Supplementary file 2 — Table S1. Two-by-two table data for symptoms and signs in relation to skin infections. (PPTX 38 kb) [file 12877_2019_1061_MOESM2_ESM.pptx]

## Slide 1
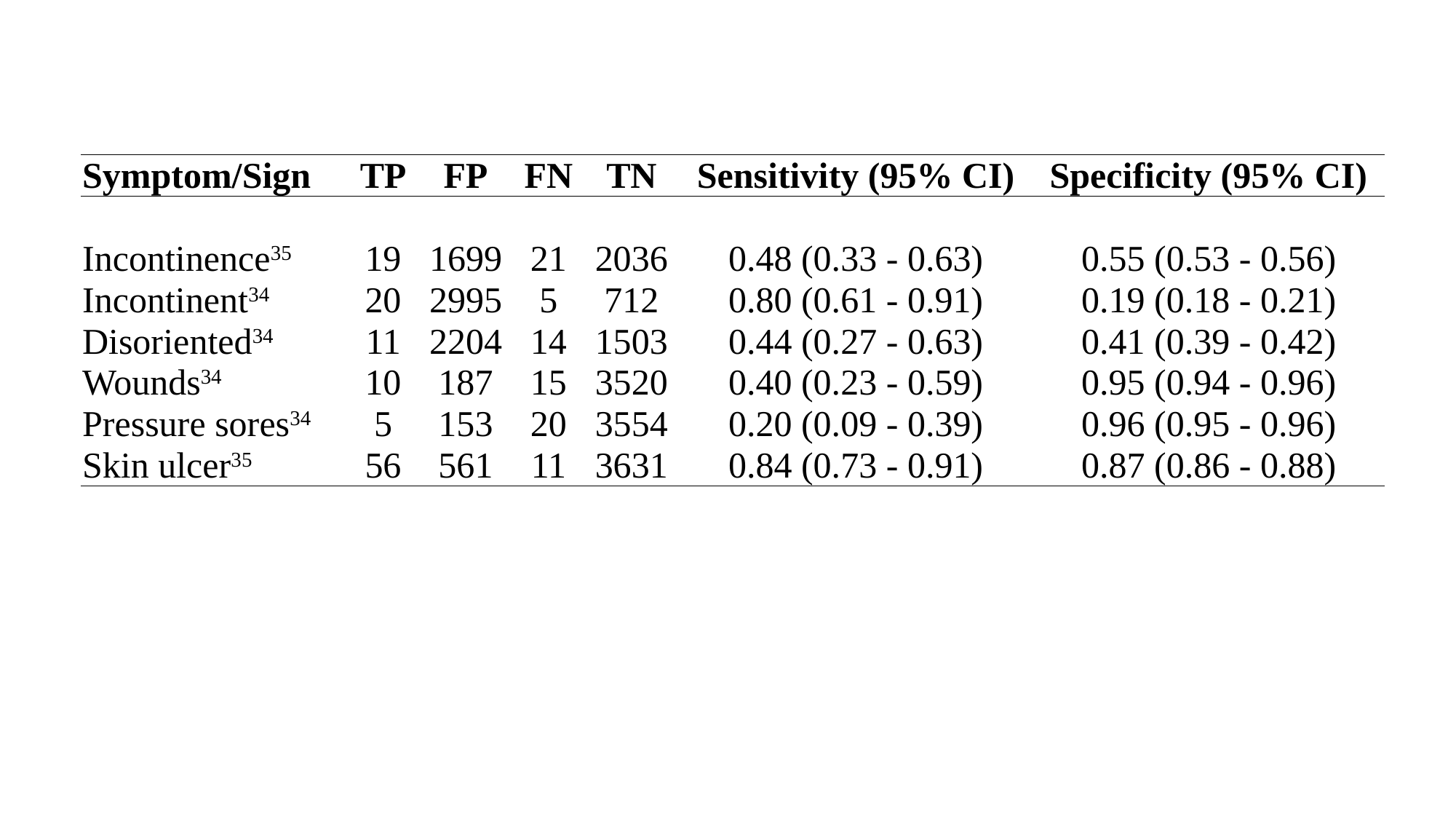

| Symptom/Sign | TP | FP | FN | TN | Sensitivity (95% CI) | Specificity (95% CI) |
| --- | --- | --- | --- | --- | --- | --- |
| | | | | | | |
| Incontinence35 | 19 | 1699 | 21 | 2036 | 0.48 (0.33 - 0.63) | 0.55 (0.53 - 0.56) |
| Incontinent34 | 20 | 2995 | 5 | 712 | 0.80 (0.61 - 0.91) | 0.19 (0.18 - 0.21) |
| Disoriented34 | 11 | 2204 | 14 | 1503 | 0.44 (0.27 - 0.63) | 0.41 (0.39 - 0.42) |
| Wounds34 | 10 | 187 | 15 | 3520 | 0.40 (0.23 - 0.59) | 0.95 (0.94 - 0.96) |
| Pressure sores34 | 5 | 153 | 20 | 3554 | 0.20 (0.09 - 0.39) | 0.96 (0.95 - 0.96) |
| Skin ulcer35 | 56 | 561 | 11 | 3631 | 0.84 (0.73 - 0.91) | 0.87 (0.86 - 0.88) |
